# Prediction of Certain Well-Characterized Domains of Known Functions within the PE and PPE Proteins of Mycobacteria

**DOI:** 10.1371/journal.pone.0146786

**Published:** 2016-02-18

**Authors:** Rafiya Sultana, Karunakar Tanneeru, Ashwin B. R. Kumar, Lalitha Guruprasad

**Affiliations:** School of Chemistry, University of Hyderabad, Hyderabad, 500046, India; University of Queensland, AUSTRALIA

## Abstract

The PE and PPE protein family are unique to mycobacteria. Though the complete genome sequences for over 500 *M*. *tuberculosis* strains and mycobacterial species are available, few PE and PPE proteins have been structurally and functionally characterized. We have therefore used bioinformatics tools to characterize the structure and function of these proteins. We selected representative members of the PE and PPE protein family by phylogeny analysis and using structure-based sequence annotation identified ten well-characterized protein domains of known function. Some of these domains were observed to be common to all mycobacterial species and some were species specific.

## Introduction

Tuberculosis (TB) caused by *Mycobacterium tuberculosis* (Mtb), remains a major global health problem and one of the main causes of death around the world [1]. About one third of the world’s population has latent TB infection. TB kills about two million people annually and is the second leading cause of death from an infectious disease worldwide, after the human immunodeficiency virus (HIV) [2,3]. As a result of reduced immunity in HIV patients, there is a greater risk of infection with TB [4,5] and significant increase in number of deaths. Despite the availability of effective short course chemotherapy, Directly Observed Treatment Short (DOTS) and the *Mycobacterium bovis* Bacille de Calmette et Guérin (BCG) vaccine, the tubercle bacillus continues to be naturally resistant to many antibiotics, making the treatment difficult [6,7]. Patients develop drug resistance resulting in the resurgence of multiple drug resistance (MDR) and extreme drug resistant (XDR) TB [8].

The complete nucleotide sequence of Mtb H37Rv strain comprising ~4,000 genes, contains two new gene families; PE and PPE, accounting for ~10% of the total genome [9]. These proteins are characterized by highly conserved N-terminal domains with approximately 110 and 180 amino acid residues, respectively. The names PE and PPE for these proteins are due to the presence of amino acid sequence motifs Pro-Glu and Pro-Pro-Glu, respectively towards the N-terminus. These proteins are proposed to be a source of antigenic variation and responsible for virulence of pathogen. Subsequently, the complete sequencing of several strains of mycobacterial genomes [10,11,12,13,14,15] identified the presence of variable numbers of PE and PPE genes. Single nucleotide polymorphisms were observed to be greater in these genes compared to the non-PE and non-PPE genes. These genes were proposed as possible vaccine candidates [9,16,17]. The PE and PPE genes were mostly arranged in a unique regulon with the PE genes located upstream to the PPE genes and scattered throughout the genome [18]. The PE and PPE genes are known to be present in pathogenic and non-pathogenic mycobacteria but have not yet been identified in non-mycobacterial species [19,20,21]. A strong evolutionary selection for PE and PPE proteins in the pathogenic mycobacteria has been reported since their expansion is linked to the ESAT-6 gene clusters that has role in immuno-pathogenesis [22].

The functions of only few PE and PPE proteins are so far known. The PE and PPE proteins are highly polymorphic and localized to the cell wall and have immunological role. Comparative analysis of the PE and PPE families in Mtb H37Rv (virulent) and H37Ra (avirulent strain) revealed genetic differences in several single nucleotide variations, insertions and deletions [23]. Comparative genomics of the *M*. *avium* complex members revealed several polymorphisms in PE and PPE family members and the presence of some unique members in PPE family that have been implicated for applications in diagnostics [24].

Among functional characterization of some of the PE and PPE proteins, it has been shown that the N-terminal PE domain of PE_PGRS33 (Rv1809) is necessary for protein localization to the cell wall in *M*. *marinum* and *M*. *tuberculosis* [25,26]. In LipY and PE_PGRS30 (Rv1651c), the C-terminus encodes the lipase activity of the protein. Both PE and PPE domains contain a signal required for secretion of LipY by the ESX-5 system and these domains are proteolytically removed upon translocation [27,28]. Studies on the enzymatic role of the PE domain in LipY revealed that PE domain down-regulates the enzyme activity of LipY, but does not effect its thermal stability [29]. Studies on the antigenic properties of operonic PE25 (Rv2431), PPE41 (Rv2430) and the complex PE25/PPE41 indicated that the PPE41 and the PE25/PPE41 complex induced significant B cell response compared to the PE25 protein [18,30]. The up-regulation of PPE32 (Rv1808) in many conditions defines its role in the host innate immune response [31,32]. The PE_PGRS63 (Rv3097c) gene is highly expressed 24 hours post-infection in murine macrophage cell lines [33] and higher expression of PE/PPE genes Rv0977, Rv1361c and Rv1840c in human macrophages upon infection have been reported [34]. Functional studies of PE and PPE family members in *M*. *tuberculosis* have reported their localization in cell wall, cytosol and membrane, and their functions have been implicated in cell wall, virulence, detoxification, adaptation, insertion sequences, lipid metabolism, intermediary metabolism, respiration and cell processes [35].

Despite the availability of the complete Mtb genome sequence very few PE, PPE proteins have been structurally and functionally characterized. Computational methods may precede the selection of proteins for wet-lab experimental validation of their structure and activity. Structure-based functional annotation of proteins has been more useful than sequence-based comparisons alone [36,37], as the protein fold and conservation of active site residues are determinants of molecular function. Therefore, in the absence of experimental structures, computer-based protein modeling methods may be employed to predict the structure and possible function [37,38,39].

Large proteins often contain domains (comprising usually >50 amino acid residues) that are known to be independent folding units, irrespective of their location along the protein sequence [40]. Our previous studies on the sequence analysis of PE and PPE proteins from Mtb H37Rv strain identified a 225 amino acid residue conserved domain. This PE-PPE domain (Pfam ID: PF08237) was observed in the C-terminus of some PE and PPE proteins [41]. Bioinformatics analyses identified a serine α/β hydrolase fold with a pentapeptide sequence motif GxSxG/S for this domain and conserved Ser, Asp and His catalytic triad residues characteristic of lipase, esterase and cutinase activities [42]. Subsequent experiments confirmed that the PE-PPE domain of PE16 (Rv1430) exhibited esterase activity [43]. Recently, Bharathy and Suguna identified and solved the three-dimensional crystal structure of an aspartic proteinase-like domain observed in the C-terminal region of the PE_PGRS16 (Rv0977) protein [44].

In the present work, we have analyzed the sequences of PE and PPE proteins from several mycobacterial species. Our results provide clues for the fold and possible functions for ten well-characterized domains predicted in some PE and PPE proteins that provide the rationale for experimental validation.

## Materials and Methods

### Sequence searches—PSI-BLAST

The amino acid sequences corresponding to the PE and PPE proteins from Mtb H37Rv strain were obtained from the NCBI databank (http://www.ncbi.nlm.nih.gov/). Iterative and reciprocal searches corresponding to the PE and PPE regions in these proteins were performed using the PSI-BLAST program (www.ncbi.nlm.nih.gov/BLAST/) against the protein sequences from 60 mycobacterial species. The PSI-BLAST program detects related proteins by deriving a position specific scoring matrix (PSSM) from multiple sequence alignment of proteins that are detected above a given threshold score [45]. The results obtained were manually inspected to confirm the protein family.

### Selection of non-redundant proteins—CD-HIT

The large dataset of mycobacterial proteins obtained from the homology searches had a high percentage of redundant sequences. We used the CD-HIT program [46] that can efficiently handle huge datasets containing millions of protein sequences, in order to remove redundant proteins and short-list set of protein sequences based on user-defined percentage sequence identity cut-off value (http://weizhong-lab.ucsd.edu/cdhit_suite/cgi-bin/index.cgi).

### Multiple sequence alignment—ClustalX

The mycobacterial PE and PPE protein sequences obtained earlier were aligned separately using the multiple sequence alignment program ClustalX 2.1. It uses a heuristic pairwise progressive sequence alignment method to generate a dendrogram. The dendrogram was used to construct the multiple sequence alignment [47]. The parameters used for multiple sequence alignment were; “10” for gap opening penalty, “0.2” for gap extension and "Gonnet Series" was chosen for protein weight matrix.

### Phylogeny analysis—MEGA5

Phylogenetic trees were generated for PE and PPE proteins using the draw tree clustering option in ClustalX based on the neighbor joining clustering algorithm. The phylogenetic tree was viewed and analyzed using Mega 5.0. MEGA5 is a collection of maximum likelihood (ML) analyses for inferring evolutionary trees, selecting best-fit substitution models for both nucleotides or amino acids, inferring ancestral states and estimating evolutionary rates [48]. The representative proteins based on these phylogenetic trees were selected for further analysis.

### Protein fold recognition—Phyre2

The non-PE and non-PPE regions in the PE and PPE proteins were selected in order to identify functional domains. Phyre2 (http://www.sbg.bio.ic.ac.uk/phyre2) comprises a suite of tools that can predict and analyze protein structure, function and mutations [49]. Phyre2 is particularly useful to recognize the protein folds of distantly related sequences and uses advanced methods for constructing three-dimensional model. The results of the model obtained for a given protein sequence can be interpreted based on percentage confidence, percentage sequence coverage, sequence alignment with the target structure, secondary and tertiary structure of the models, domain composition, conservation of the active site and model quality.

## Results and Discussion

Mycobacterial species are known to comprise variable numbers of PE and PPE family proteins. Several variations in their protein sequences have been attributed to synonymous and non-synonymous single nucleotide polymorphism (SNPs), in-frame deletions and insertions, resulting in their altered physico-chemical properties [23,24,50]. These features indicate the observed differences between the mycobacterial species. The N-terminal PE and PPE domains, and the Gly-rich regions in these families of proteins provide limited clues on their possible structure and function. The structure-based annotation of the protein folds are more useful, which have therefore been applied in the present work using Phyre2 program that has also been useful to assign a possible function. The list of mycobacterial species for the analysis of structure and function corresponding to the PE and PPE protein families studied in this work are shown in the supplementary data (S1 Table). The protein fold templates identified with ‘high’ confidence and described as "certain" according to the program and corresponding to significant sequence coverage over whole length of the protein were selected as the probable fold. The three-dimensional models based on the templates identified and the alignments of the sequence to each of the templates produced by Phyre2 were manually inspected to examine whether the catalytic residues and the cofactor binding residues were also conserved. In situations where such conservation was confirmed, the corresponding domains were assigned as the protein fold. We discuss below the different domains that were identified in the PE and PPE proteins.

### Hydrolase domain

The PE and PPE proteins from several mycobacterial species (*M*. *tuberculosis*, *M*. *bovis*, *M*. *africanum*, *M*. *canettii*, *M*. *marinum*, *M*. *kansasii*, *M*. *liflandi*, *M*. *heckeshornense*, *M*. *bovis BCG strain*, *M*. *asiaticum*, *M*. *caprae*, *M*. *nebraskense*, *M*. *gordonae*, *M*. *haemophilum*, *M*. *lentiflavum*, *M*. *simiae*, *M*. *triplex*, *M*. *sinense*, *M*. *arupense*, *M*. *heraklionense*, *M*. *neworleansense*) that were predicted to comprise a conserved C-terminal domain with α/β hydrolase fold are shown in the supplementary data (S1 Appendix). This domain was modelled on the crystal structures of two templates comprising msmeg_6394 from *M*. *smegmatis* str. MC2 155 (PDB_ID: 3AJA:A) and a putative esterase from *Staphylococcus aureus* (PDB_ID: 3D7R:B). The template structures represent hydrolase family which exhibit an overall α/β hydrolase fold with central β-sheet flanked by α-helices on either side of the sheet and has the functional characterization of a lipase. Most hydolase family proteins are characterized by a beta-sheet core made up of five to eight β-strands connected by α-helices forming an α/β/α sandwich with a conserved pentapeptide sequence motif GxSxG, and Ser, Asp and His as the catalytic residues [51].

We previously identified this domain in the C-terminal region of 8 PE and PPE proteins (Rv0151c, Rv0152c, Rv0159c, Rv0160c, Rv1430, Rv1800, Rv2608 and Rv3539) in *M*. *tuberculosis* H37Rv strain and was termed ‘PE-PPE domain’ [41]. Subsequently, we modelled this domain, characterized the active site, lid insertion close to the active site, the oxyanion hole required for function and predicted that this domain would specifically possess esterase/lipase/cutinase activity [42]. Further, we carried out wet-lab experimental studies using biochemical assays and mutational analyses and confirmed that the purified full-length Rv1430 protein and its ‘PE-PPE domain’ possesses esterase activity and hydrolyses short to medium chain fatty acid esters with highest specific activity for p-nitrophenyl caproate [43].

### Aspartic proteinase domain

The PE proteins from mycobacterial species (*M*. *tuberculosis*, *M*. *bovis*, *M*. *caprae*, *M*. *africanum*, *M*. *caprae*, *M*. *orygis*, *M*. *canettii*, *M*. *gordonae*) predicted to comprise the aspartic proteinase domain are shown in the supplementary data (S2 Appendix). This domain was reported in the PE_PGRS16 (Rv0977) protein and its three-dimensional crystal structure was solved (PDB_ID: 4EHC) [44]. The aspartic proteinase domain has low overall sequence similarity to HIV proteinase with a characteristic pepsin-fold and catalytic site architecture. The overall fold comprises a six stranded β-sheet located at the centre formed by the contribution of 3 strands each from N- and C-terminal domains. On either side of the central region, β-sheet rich regions and two α-helices connected by loops that harbor the conserved DTG motifs were located. These motifs are essential for the peptide hydrolysis function of this enzyme. In this work, the fold for some of the PE proteins, for instance, WP_015355774.1, WP_036418539.1 and WP_031667305.1 (refer S2 Appendix for detailed list) comprising a C-terminal 280 amino acid domain was predicted as the aspartic proteinase domain. This domain in the mycobacterial PE proteins was modeled on the crystal structure of PDB_ID: 4EHC_A. The models comprise the two conserved DT(S)G motifs characteristic of aspartic proteinases. In the peptide hydrolysis reaction, one of the aspartic acid residues from the conserved motifs acts as a general base and the other acts as a general acid followed by the nucleophilic attack of a catalytic water molecule [52]. Typically aspartic proteinases hydrolyse a peptide bond between hydrophobic residues. For example, renin is an aspartic proteinase that cleaves angiotensiogen to a decapetide angiotensin with high specific cleavage between Leu-Leu bond [53]. Several of these proteins are drug targets, for example, HIV proteinase in AIDS and renin in hypertension [54].

### Glucosyl-3-phosphoglycerate phosphatase domain

**Fig 1:**
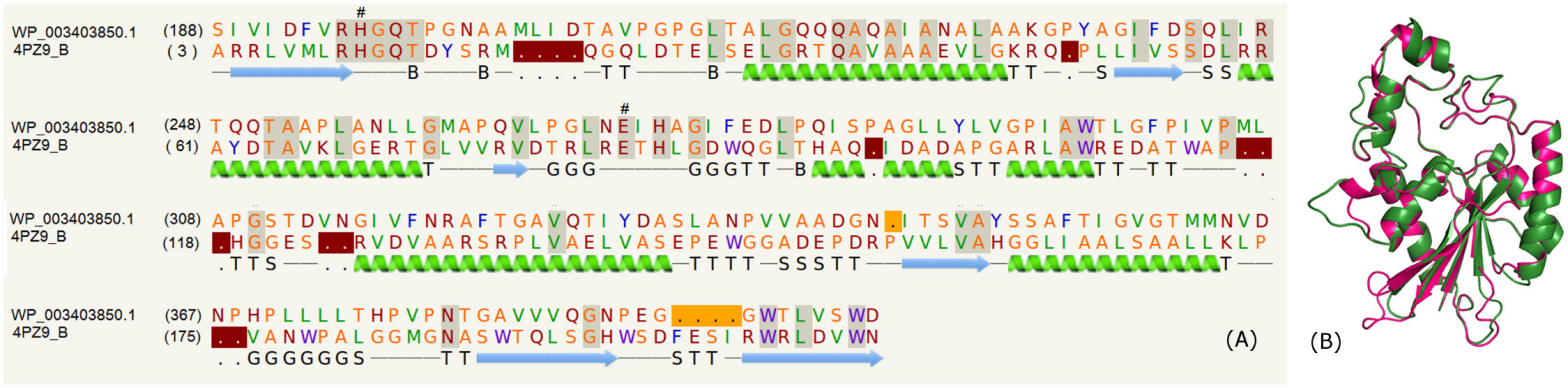
Glucosyl-3-phosphoglycerate phosphatase domain. (A) Sequence alignment of model WP_003403850.1 and template PDB_ID: 4PZ9:B indicating the catalytic residues (#). In all the pair-wise sequence alignments, identical residues are indicated in gray shaded regions, deletions in the template sequence is indicated as dots in brown shaded region, deletions in the query sequence is indicated as dots in yellow shaded region. (B) Structure alignment of model (green) and template (pink).

The list of PE proteins from mycobacterial species (*M*. *tuberculosis*, *M*. *bohemicum*, *M*. *haemophilum*, *M*. *canettii*, *M*. *kansasii*, *M*. *africanum*, *M*. *bovis*, *M*. *marinum*, *M*. *liflandii*, *M*. *xenopi*, *M*. *heckeshornense*, *M*. *gordonae*) comprising the glucosyl-3-phosphoglycerate phosphatase domain is shown in the supplementary data (S3 Appendix). Some of these PE proteins, for instance, WP_003403850.1, WP_015357328.1 and CPR12073.1 recognized the template PDB_ID: 4PZ9:B corresponding to the mycobacterial glucosyl-3-phosphoglycerate phosphatase Rv2419c [55]. This enzyme consists of a single domain made up of a central β-sheet flanked by α-helices on either side and is known to catalyze the second step in the biosynthesis of methylglucose lipopolysaccharides (MGLPs) pathway. The synthesis of mycolic acids, which forms an important lipid component of the mycobacterium cell wall, is regulated by MGLPs. The alignment of the sequence to the template (~21% identity) is shown in Fig 1A and highlights the regions of secondary structure. The corresponding structure alignment of the model and template is shown in Fig 1B and suggests the overall similarity in the protein fold.

In the crystal structure (PDB_ID: 4QIH), the active site is located in a positively charged cleft situated above a central β-sheet. Unambiguous electron density for a vanadate ion covalently bound to His11 (numbering according to PDB_ID: 4PZ9) mimicking the phosphohistidine intermediate and acetate ion was observed. In WP_003403850.1 too, the catalytic residues His11 and Glu84 are conserved (Fig 1A) and most of the ligand binding residues close to the acetate and vanadate, for instance, Arg10, His11, Asn17, Gln23, Arg60, Glu84, His159 and Leu209 which are important for enzymatic activity are conserved, except Gln23 which was replaced by Thr and His159 by Tyr. According to the structure analyses, most residues being conserved, especially residues close to vanadate, suggests that the protein function is also likely to be conserved.

### Laminaripentaose-producing beta-1,3-glucanase domain

**Fig 2:**
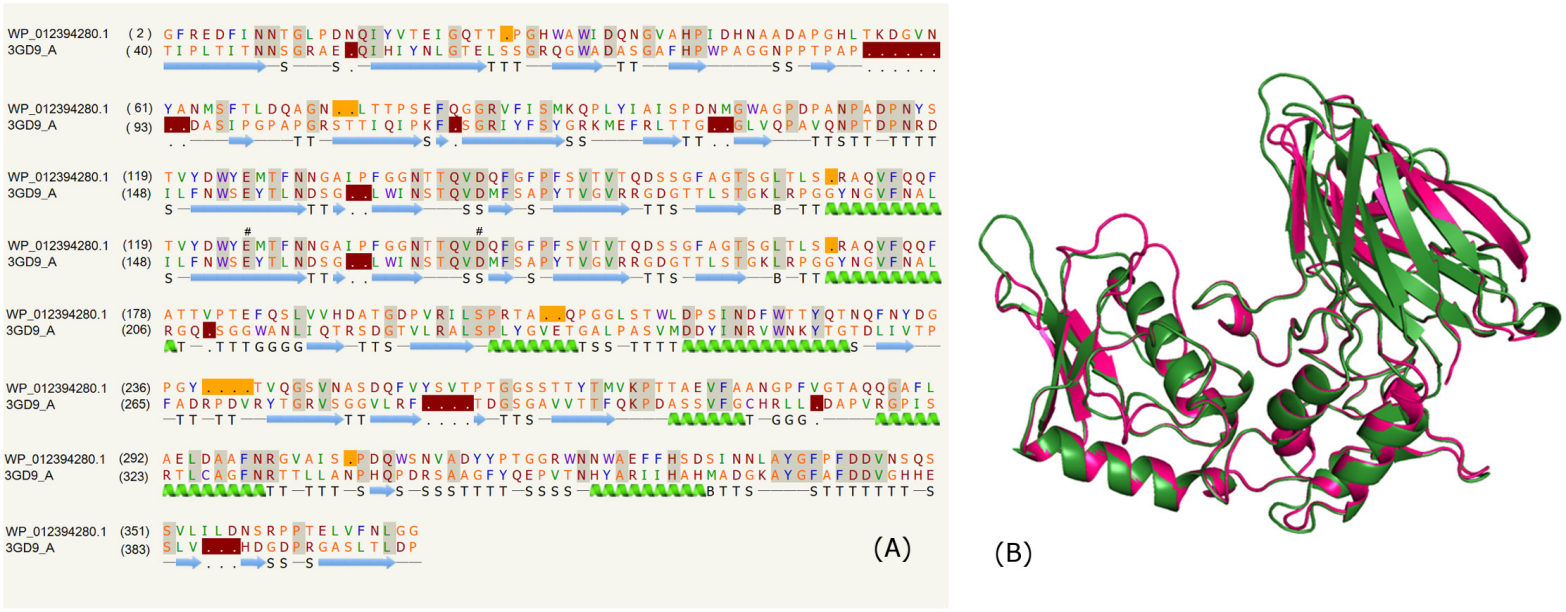
Laminaripentaose-producing beta-1,3-glucanase domain. (A) Sequence alignment of model WP_012394280.1 and template PDB _ID: 3GD9:A indicating the catalytic residues (#). (B) Structure alignment of model (green) and template (pink).

The list of PE proteins from mycobacterial species (*M*. *marinum*, *Mycobacterium sp*. *012931*, *M*. *ulcerans str*. *Harvey*) comprising the laminaripentaose-producing beta-1,3-glucanase (LPHase) domain is shown in the supplementary data (S4 Appendix). The PE protein, for instance, WP_012394280.1 from *M*. *marinum* C-terminal region recognized the template PDB_ID: 3GD9:A corresponding to the crystal structure of LPHase in complex with laminaritetraose [56]. Glycoside hydrolases have been classified into families based on sequence similarity and have been further grouped into clans based on the similarity of their overall fold, active site architecture and catalytic mechanism [57,58,59]. LPHase is a glycoside hydrolase family 64 protein which cleaves a long chain polysaccharide β-1,3-glucan into specific pentasaccharide oligomers. The structure consists of a crescent-like fold; a barrel domain and a mixed (α/β) domain forming a wide-open groove between the two domains. The sequence alignment highlighting the secondary structures predicted is shown in Fig 2A and has ~24% identity. The structural overlay of the model and template is shown in Fig 2B that suggests the protein has overall similar fold and certain variable loop regions. The glycoside hydrolases are known to catalyse the hydrolysis of the glycosidic bond between two or more carbohydrates or between a carbohydrate and non-carbohydrate moiety [60]. Depending on the nature of the organism, these enzymes are associated with a variety of roles, such as degradation of biomass by cellulases, pathogenesis during the activity of influenza virus neuraminidase [61], normal cellular metabolic processes that involves the formation and breakage of glycosidic bonds [62]. Our model suggests the conservation of catalytic residues; Glu154 and Asp170 (numbering according to the PDB_ID: 3GD9) as shown in Fig 2A. Between the N and C-terminal domains, the model contains an electronegatively charged wide groove comprising several conserved residues that include the above catalytic residues and four amino acid residues; Thr156, Asn158, Trp163 and Thr167 involved in sugar binding that accommodate the laminaritetraose molecule. According to the crystal structure of LPHase (PDB_ID:3GD9), the enzyme uses a direct displacement mechanism involving Glu154 and Asp170 via acid-base catalysis to cleave β-1,3-glucan into specific α-pentasaccharide oligomer. The side chains of Thr156, Asn158 and Trp163 are known to demarcate the subsite +5 in the active site.

### Chitinase domain

**Fig 3:**
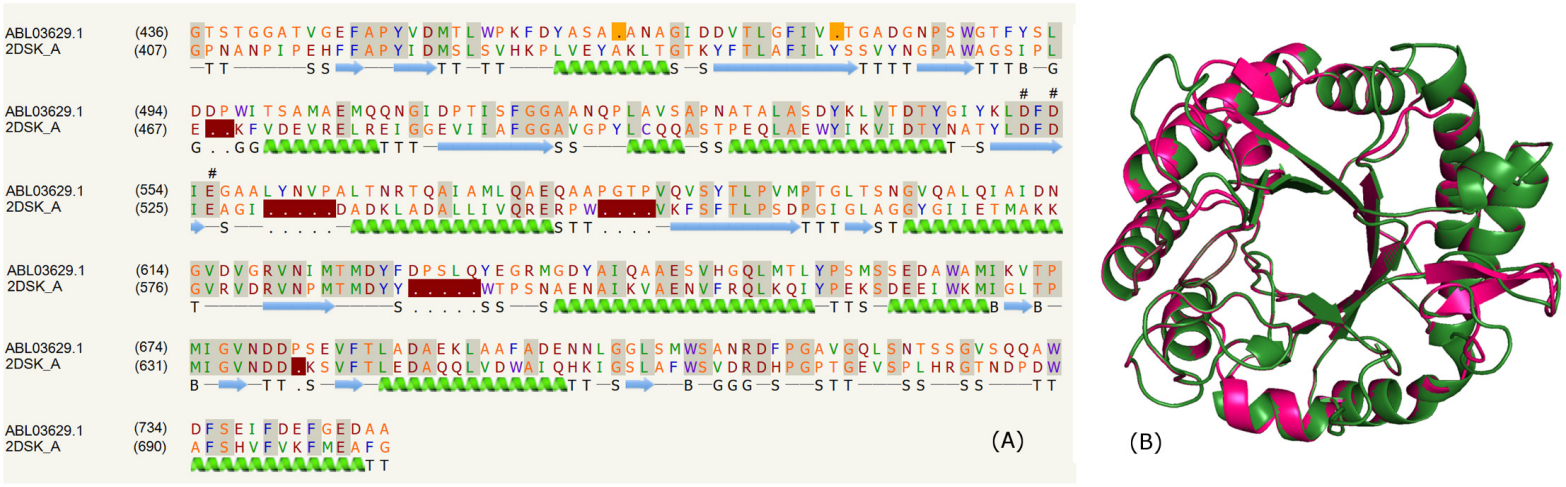
Chitinase domain. (A) Sequence alignment of model ABL03629.1 and template PDB _ID: 2DSK:A indicating the catalytic residues (#). (B) Structure alignment of model (green) and template (pink).

The list of PE proteins from mycobacterial species (*M*. *liflandii*, *M*. *marinum*, *Mycobacterium sp*. *012931*, *M*. *ulcerans str*. *Harvey*, *M*. *gastri*, *M*. *gordonae*) comprising the chitinase domain is shown in the supplementary data (S5 Appendix). Some of these PE proteins, for instance, ABL03629.1, WP_015355330.1, WP_023367572.1, WP_036414736.1 and WP_012395848.1 recognize the template PDB_ID:2DSK:A corresponding to the chitinase domain. The catalytic site residues; Asp522, Asp524 and Glu526 form the characteristic DxDxE motif observed in the crystal structure were conserved in these PE proteins as shown for one of the illustrative examples in Fig 3A that shares ~37% identity. The model was constructed on the crystal structure of the catalytic domain of chitinase from *Pyrococcus furiosus* (PDB _ID: 2DSK:A). The overall structure of this domain comprises a TIM-barrel fold with a tunnel-like active site, a common feature of family 18 chitinases. The high degree of the overall structural similarity is shown in Fig 3B. The chitinases hydrolyze chitin, a polymer of β-1,4-linked N-acetylglucosamine (GlcNAc) and classified into two families (families 18 and 19 in the CAZy database; http://www.cazy.org/) according to amino acid sequence similarity [63].

### Endoglucanase domain

**Fig 4:**
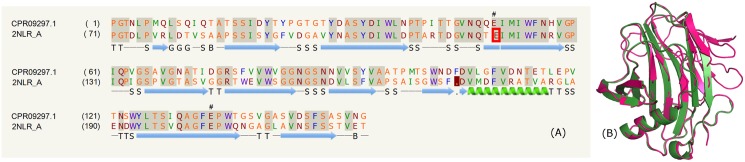
Endoglucanase domain. (A) Sequence alignment of model CPR09297.1 and template PDB_ID: 2NLR:A indicating the catalytic residues (#, red box). (B) Structure alignment of model (green) and template (pink).

The list of PE proteins from mycobacterial species (*M*. *kansasii*, *M*. *gastri*, *M*. *gordonae*, *M*. *bohemicum DSM 44277*, *M*. *asiaticum*) comprising the endoglucanase domain is shown in the supplementary data (S6 Appendix). The PE proteins, for example, MGAST_01715 and CPR09297.1 have a conserved C-terminal region. The fold for these proteins corresponds to the endoglucanases (PDB_IDs: 1OA4:A and 2NLR). These glycoside hydrolase clan GH-C group endoglucanases comprise the family 11 xylanases and family 12 cellulases, which share a jelly-roll topology. The two predominantly anti-parallel β-sheets form a long substrate-binding cleft. The catalysis of these enzymes is via a double-displacement mechanism in which a covalent glycosyl enzyme intermediate is formed and subsequently hydrolyzed with acid-base assistance, via oxocarbenium ion transition states and performs the catalysis with net retention of anomeric configuration [64,65]. Fig 4A shows the sequence alignment (~58% identity) along with the catalytic residues and the secondary structural information. The structural comparison is shown in Fig 4B. The concave surface of the larger β-sheet produces a wide substrate-binding cleft across one face of the enzyme [66]. Further, the cleft has two invariant catalytic residues Glu120 and Glu203 (amino acid numbering according to PDB_IDs: 2NLR) that point into the active site cleft from opposite sides. A long loop crossing the substrate-binding groove terminates at the reducing end, known as the “cord” and that is a common feature of all family 11 and family 12 structures. A number of residues located in the loop, importantly, Pro133 is conserved throughout clan GH-C members. A possible loop movement upon substrate binding has been speculated [66].

### Carbohydrate binding domain

**Fig 5:**
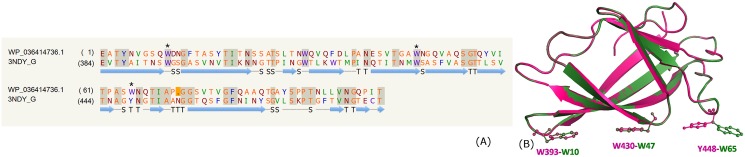
Carbohydrate binding domain. (A) Sequence alignment of model WP_036414736.1 and template PDB_ID: 3NDY:G indicating aromatic residues (*) from hydrophobic strip. (B) Structure alignment of model (green) and template (pink).

The list of PE proteins from mycobacterial species (*M*. *kansasii*, *M*. *gastri*, *Mycobacterium sp*. *012931*, *M*. *bohemicum DSM 44277*) comprising the carbohydrate binding domain is shown in the supplementary data (S7 Appendix). In the cellulose degradation process, the binding of the cellulolytic enzyme is mediated by carbohydrate binding domain (CBD) which typically comprises ~100 amino acid residues [67]. CBDs are separated from the catalytic domain by a short amino acid linker region and the enzymatic degradation is carried out by the cellulolytic domain [68,69]. From our analysis, we observed that the CPR09297.1 comprising an endoglucanase domain described above has one CBD, while ETW25608.1 has two consecutive CBDs separated by 87 amino acids. The two proteins with CBD domains are linked with C-terminal glycosyl hydrolase 12 (GH12) family domains discussed above. Likewise, another PE protein; WP_036414736.1, comprises a CBD that is linked to a glycosyl hydrolase 18 (GH18) family domain. The three-dimensional structures of these CBDs were modelled on the CBD domain present in the endoglucanase D from *Clostridium cellulovorans* (PDB_ID: 3NDY). The sequence alignment (~35% identity) is shown in Fig 5A. The alignment demonstrates that the secondary structure is mainly comprised of beta-strands. The structural overlay of model with template is shown in Fig 5B which reveal eight major beta-strands that fold into a beta-sheet structure. Three conserved hydrophobic amino acids (Trp, Trp, Trp/Tyr) ‘strip’ are located on loops connecting the beta-strands in the model and the template as shown in Fig 5B. The pi-electron dense aromatic rings of the ‘strip’ contact the cellulose hydrophobic region and drive the enzymes to perform their catalytic functions [70,71].

### Cytochrome P450 domain

**Fig 6:**
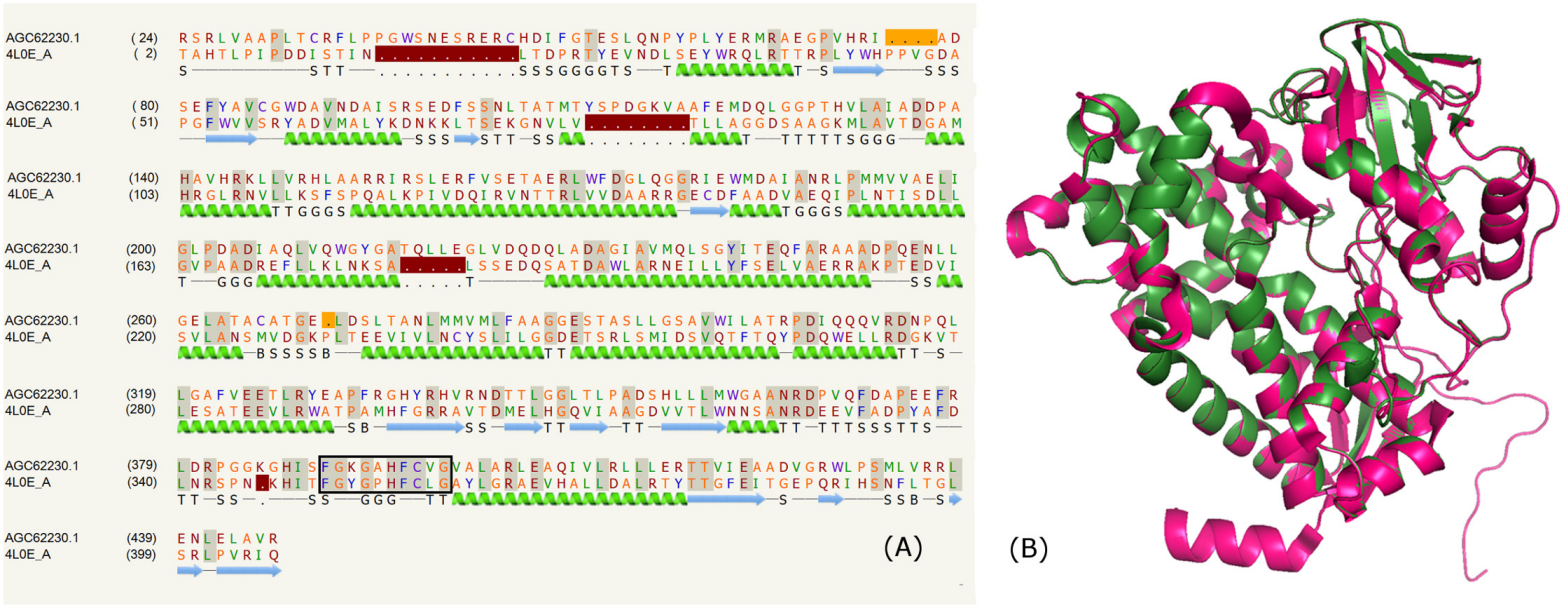
Cytochrome P450 domain. (A) Sequence alignment of model AGC62230.1 and template PDB _ID: 4L0E:A indicating consensus heme binding decapeptide motif in box. (B) Structure alignment of model (green) and template (pink).

The 1761 amino acid PE protein AGC62230.1 from *M*. *liflandii* 128FXT comprises a 500 amino acid C-terminus domain. The fold for this domain was recognized as the cytochrome P450 from *Streptomyces sp*. *Acta* 2897 (PDB_ID:4L0E) [72]. Cytochrome P450s are a class of heme cofactor binding proteins and found in all domains of life. The high diversity in sequences and functions resulted in an expanded family of cytochrome P450s. They catalyse a variety of reactions, for instance, carbon heteroatom oxygenation, dealkylation, epoxidation, aromatic hydroxylation, reduction and dehalogenation [73]. A unique consensus sequence motif; ‘FXXGXXXCXG’ is present in all cytochrome P450s and located between helices K and L that forms a heme binding decapeptide loop [74]. This motif was also observed in the PE protein AGC62230.1 from *Mycobacterium liflandii* 128FXT (Fig 6A).

The non-ribosomal peptide synthetases (NRPSs) are involved in the synthesis of diverse peptides known as non-ribosomally synthesised peptides (NRPs) [75]. One of the prominent modifications found in NRPs is the β-hydroxylation of various amino acid residues including the hydroxylation of non-activated C-H bonds [76] that are catalysed by cytochrome P450 enzymes. The cytochrome P450 encoded (sky32) is associated with the skyllamycin biosynthesis gene cluster. The cyclodepsipeptide skyllamycin A isolated from streptomyces is an inhibitor of the platelet derived growth factor signaling pathway [77]. The crystal structure of sky32 (PDB_ID:4L0E) is responsible for the β-hydroxylation of three separate amino acids at positions 5 (β-hydroxyphenylalanine), 7 (β-hydroxy-OMe-tyrosine), and 11 (β-hydroxyleucine). The sequence alignment (~23% identity) corresponding to the cytochrome P450 domain in the above PE protein and sky32 mainly comprising helices is shown in Fig 6A. The comparison of the overall fold shown in Fig 6B reveals the high degree of structural similarity.

### Beta-propeller

**Fig 7:**
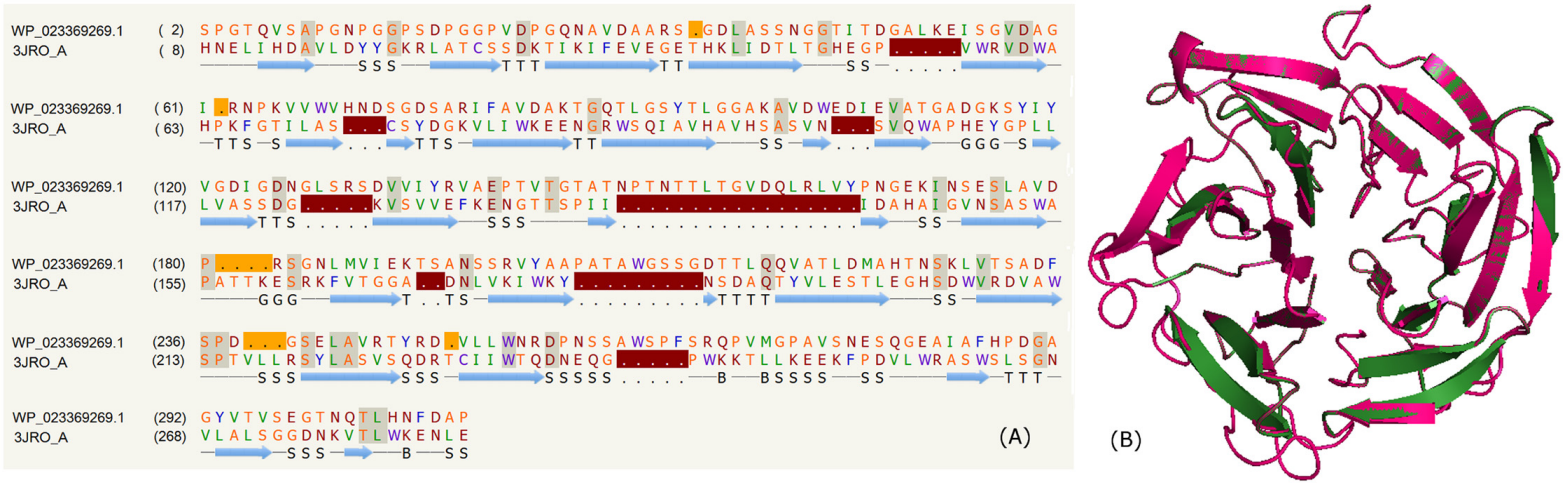
Beta-propeller. (A) Sequence alignment of model WP_023369269.1 and template PDB_ID: 3JRO:A. (B) Structure alignment of model (green) and template (pink).

We earlier reported certain PE family proteins to comprise YVTN repeats [41]. These repeats contain 40–45 amino acid residues present in tandem along the protein sequence and located towards the C-terminus. In this work, we have identified several PE proteins from mycobacterial species (*M*. *tuberculosis*, *M*. *bovis*, *M*. *africanum MAL020173*, *M*. *caprae*, *M*. *orygis 112400015*, *M*. *canettii CIPT 140070010*, *M*. *haemophilum*, *M*. *marinum*) that contain the above repeat as shown in the supplementary data (S8 Appendix). Some of these PE proteins, for example, WP_023369269.1, CCP43730.1, WP_013988789.1 and WP_013988787.1 were modelled on the crystal structures of nitrous oxide reductase from *Pseudomonas nautical* (PDB_ID: 1QNI:E) [78], nitrous oxide reductase from *P*. *denitrificans* (PDB_ID: 1FWX:B) [79], cytochrome cd1 nitrite reductase (PDB_ID: 1GQ1:B) [80] and nup84-nup145c-sec13 (PDB_ID: 3JRO:A) [81]. These diverse proteins comprise a 6–8 bladed beta-propeller fold. Typically, beta- propellers contain 4–8 blades that are arranged circularly around a central axis [82]. Several other diverse ~40–45 amino acid repeats, such as, WD, YWTD, etc., are known to be present in tandem and fold as beta-propellers [83]. The proteins containing the beta-propellers are associated with diverse functions such as transport, hydrolases, transferases, sugar and cofactor binding proteins, cell surface proteins, lyases and isomerases [83]. In some cases, the active site is present in the loops that connect the tandem blades. For example, in the crystal structure of influenza neuraminidase (PDB_ID: 1BJI), the active site is located in the region connected by several loops [84]. The sequence alignment (~13% identity) corresponding to the PE protein; CMB12570.1 and its template is shown in Fig 7A. The structural comparison of the model and template is shown in Fig 7B. This protein represents a 6 bladed beta-propeller.

### Beta-helix

**Fig 8:**
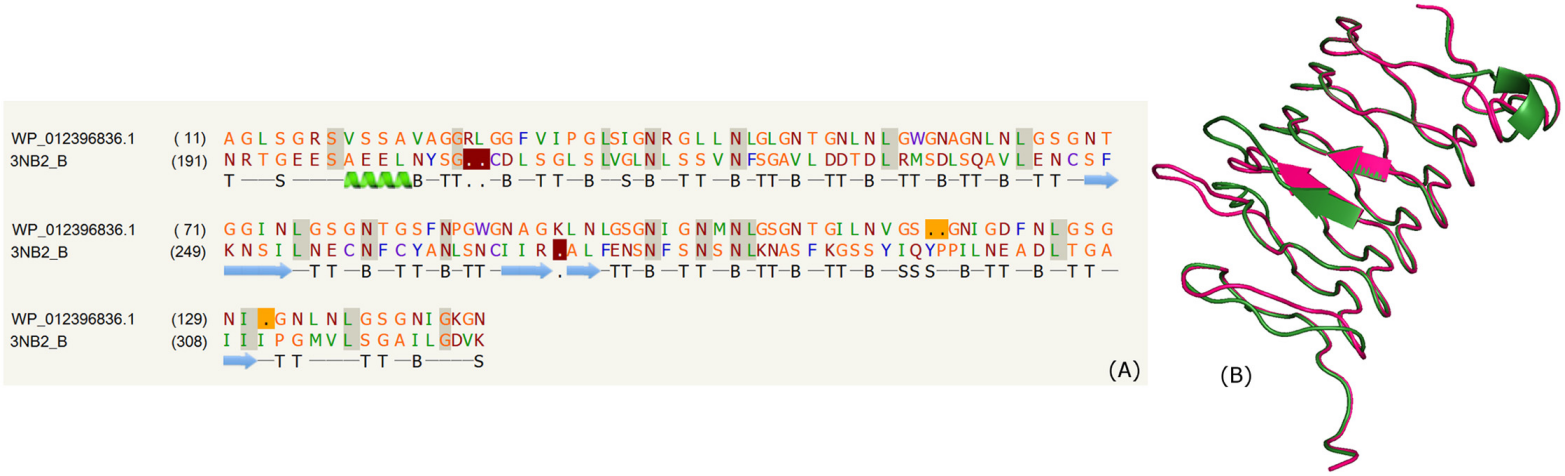
Beta-helix. (A) Sequence alignment of model WP_012396836.1 and template PDB_ID: 3NB2:B. (B) Structure alignment of model (green) and template (pink).

The beta-helix predicted for the PPE proteins from mycobacterial species (*M*. *tuberculosis*, *M*. *kansasii*, *M*. *asiaticum*, *M*. *gastri*, *Mycobacterium sp*. *012931*, *M*. *marinum*, *M*. *canettii*, *M*. *gordonae*, *M*. *ulcerans*, *M*. *bovis*, *M*. *orygis*, *M*. *liflandii*) is shown in the supplementary data (S9 Appendix). Several PPE proteins are characterized by Gly-rich pentapeptide sequence repeats. Some of the PPE proteins (WP_049959014.1, WP_049959014.1 and WP_012396836.1) were modelled on the N-terminal domain of a ubiquitin ligase (PDB _ID: 3NB2:B) as the template. The template structure contains two structural domains: an N-terminal four stranded beta-helix domain made up of penta-peptide sequence repeats and a C-terminal α-helical catalytic domain [85,86]. Beta-helices were initially identified in the crystal structure of pectate lyase [87] and their functions, such as, polysaccharide lyases, cellulose or acid sugar binding lyases have been reviewed [88]. The beta-helix forms a helical pattern due to the hydrogen bonds between parallel beta sheets and can form two/three/four beta-stranded helices. The sequence alignment (~15% identity) predicted to mainly comprise the beta-strands is shown in Fig 8A and structure superposition on the template for this domain predicted in WP_012396836.1 is shown in Fig 8B.

### Acetyl hydrolase / cutinase domain

**Fig 9:**
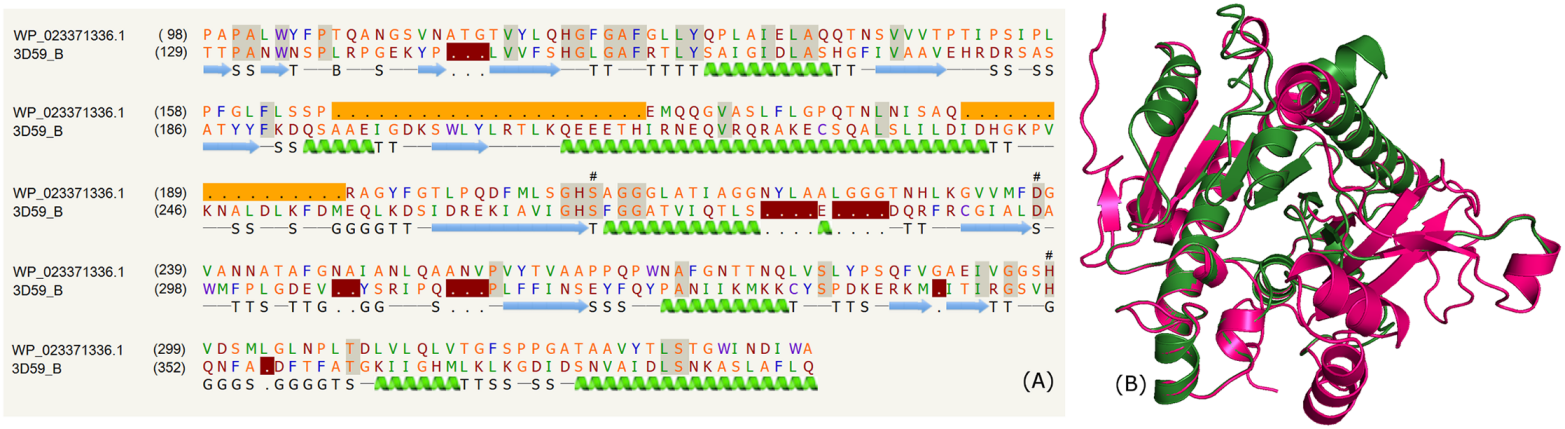
Acetyl hydrolase/cutinase. (A) Sequence alignment of model WP_023371336.1 and template PDB _ID: 3D59:B indicating the catalytic residues (#). (B) Structure alignment of model (green) and template (pink).

The list of PE proteins from mycobacterial species (*M*. *bohemicum DSM 44277*, *M*. *marinum*, *M*. *liflandii*, *Mycobacterium sp*. *012931*, *M*. *kansasii*, *M*. *asiaticum*, *M*. *gordonae*) predicted to contain the acetyl hydrolase / cutinase domain is shown in the supplementary data (S10 Appendix). Some of these proteins were modeled, for instance, WP_023371336.1, WP_015356298.1, WP_036353055.1 and CPR09862.1. The crystal structures of human plasma platelet activating factor acetyl hydrolase (PDB_IDs: 3D5E, 3D59:B) were used as templates in the modeling procedure. These structures have a α/β-hydrolase fold containing a catalytic triad of Ser, His and Asp. The alignment of the sequences (~15% identity) is shown in Fig 9A. The structural comparison for the PE protein; WP_023371336.1 with template is shown in Fig 9B. The location of the catalytic residues Ser273, Asp296 and His351 (numbering according to PDB_ID: 3D59) are shown in Fig 9A.

### Transmembrane domain

**Fig 10:**
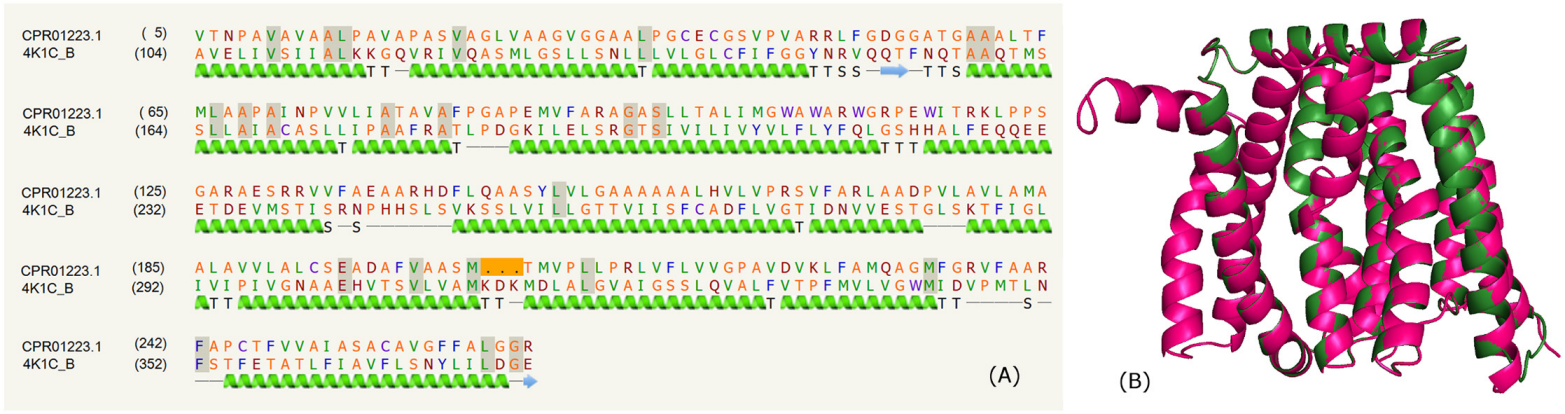
Transmembrane domain. (A) Sequence alignment of model CPR01223.1 and template PDB _ID: 4K1C:B. (B) Structure alignment of model (green) and template (pink).

Some PPE family proteins; WP_036437315.1, WP_036444818.1 and CPR01223.1 have been predicted to comprise transmembrane helices with large stretches of intervening sequences suggesting these as membrane proteins. We modeled, one of the above proteins, CPR01223.1, a *M*. *bohemicum* protein on the crystal structure of a eukaryotic calcium/proton exchanger (PDB_ID: 4K1C). The sequence alignment that shares ~9% identity and predicted secondary structure is shown in Fig 10A, suggesting this protein is mainly composed of helices. The three-dimensional model superimposed on the template is shown in Fig 10B.

In this work, we have identified certain well-characterized domains present in the PE and PPE proteins of mycobacteria. These domains are known to be associated with a variety of functions, such as, hydrolysis of lipids (α/β hydrolase fold and acetyl hydrolase), hydrolysis of carbohydrates (chitinase, endoglucanase and laminaripentaose-producing beta-1,3-glucanase domain) and hydrolysis of proteins (aspartic proteinase). Glucosyl-3-phosphoglycerate phosphatase plays an important role in the synthesis of mycobacterial cell wall components and cytochrome P450 domain is implicated in metabolic activity. The transmembrane domains, beta-propellers, CBD and beta-helices are regulators of protein function. This suggests that some of the PE and PPE family proteins may be associated with enzymatic and regulatory roles.

The α/β hydrolase domain is present in both PE and PPE family proteins, whereas aspartic proteinase, chitinase, endoglucanase and beta-propeller domains were only detected for PE proteins. The beta-helix has been observed in PPE proteins. It was also observed that some domains such as α/β hydrolase were present in all mycobacterial species, whereas some domains, such as, chitinase and endoglucanase were specific only to certain mycobacterial species. These findings suggest that the PE and PPE family proteins co-ordinate diverse roles that are mycobacterial species dependent.

Out of several hundred diverse sequences that were analysed, we were able to predict the fold with ‘high’ confidence for ~30% proteins. The structure and function predicted for the PE and PPE proteins discussed in this work provide the rationale for validation by experimental studies.

## Conclusions

The bioinformatics analyses of several PE and PPE proteins from a number of mycobacterial species allowed us to identify the following well-characterized domains; hydrolase, aspartic proteinase, glucosyl-3-phosphoglycerate phosphatase, laminaripentaose-producing beta-1,3-glucanase, chitinase, endoglucanase, carbohydrate binding, cytochrome P450, beta-propeller, beta-helix, acetyl hydrolase/cutinase and transmembrane domains. Some of these domains have enzymatic roles and hydrolyse substrates, such as, proteins, lipids and carbohydrates, while some domains have a regulatory role. Further, some domains were observed to be common to several mycobacterial species, while some were present only in few mycobacterial species. Our work sheds new light on the structural and functional aspects of these important classes of mycobacterial proteins.

## Supporting Information

S1 Appendix Mycobacterial PE and PPE proteins that comprise the α/β hydrolase fold. (XLS)

S2 Appendix Mycobacterial PE proteins that comprise the aspartic proteinase domain. (XLS)

S3 Appendix Mycobacterial PE proteins that comprise the glucosyl-3-phosphoglycerate phosphatase domain. (XLS)

S4 Appendix Mycobacterial PE proteins that comprise the laminaripentaose-producing beta-1,3-glucanase domain. (XLS)

S5 Appendix Mycobacterial PE proteins that comprise the chitinase domain. (XLS)

S6 Appendix Mycobacterial PE proteins that comprise the endoglucanase domain. (XLS)

S7 Appendix Mycobacterial PE proteins that comprise the carbohydrate binding domain. (XLS)

S8 Appendix Mycobacterial PE proteins that comprise beta-propeller. (XLS)

S9 Appendix Mycobacterial PPE proteins that comprise beta- helix. (XLS)

S10 Appendix Mycobacterial PE proteins that comprise the acetyl hydrolase domain. (XLS)

S1 Table Mycobacterial species used for the structure and function analyses of the PE and PPE proteins. (DOC)
