# Supplementary material for: Prediction of Certain Well-Characterized Domains of Known Functions within the PE and PPE Proteins of Mycobacteria
Source: PLoS One. 2016 Feb 18;11(2):e0146786. doi: 10.1371/journal.pone.0146786 (PMC4758615; doi:10.1371/journal.pone.0146786)
Supplement: S1 Table — (DOC) [file pone.0146786.s011.doc]

S1 Table. Mycobacterial species analyzed for identification of domains in PE and PPE proteins

| **Mycobacterial species** | **Infection type** |
| --- | --- |
| *Mycobacterium abscessus* subsp. bolletii str. GO 06 | Opportunistic infections |
| *Mycobacterium africanum* GM041182 | Human tuberculosis in West Africa |
| *Mycobacterium avium* subsp. paratuberculosis K-10 | The causative agent of Johne’s disease in cattle and other ruminants |
| *Mycobacterium bovis* BCG str. Pasteur 1173P2 | Vaccine |
| *Mycobacterium canettii* CIPT 140010059 | Pulmonary tuberculosis |
| *Mycobacterium chubuense* NBB4 | Non-pathogenic strain, versatile hydrocarbon degrader |
| *Mycobacterium gilvum* PYR-GCK | Non-pathogenic strain, also useful in the application of bioremediation processes since they can degrade high-molecular-weight polycyclic aromatic hydrocarbons (PAHs) |
| *Mycobacterium gilvum* Spyr1 | - |
| *Mycobacterium indicuspranii* MTCC9506 M | Immunotherapeutic against leprosy and also approved as a vaccine |
| *Mycobacterium intracellulare* MOTT-64 | Causes tuberculosis in birds and pulmonary and disseminated infections in immunocompromized humans |
| *Mycobacterium leprae* Br4923 | An unculturable obligate pathogen that causes leprosy in humans |
| *Mycobacterium liflandii* 128FXT | Frog pathogen |
| *Mycobacterium marinum* M | Pathogen of fish and amphibia, is a near relative of MTB, the etiologic agent of tuberculosis in humans |
| *Mycobacterium neoaurum* VKM Ac-1815D | Synthesizes the valuable steroid precursor 4-androstene-3,17-dione as a major product from sitosterol |
| *Mycobacterium rhodesiae* NBB3 | Rare cases of peritonitis, also involved in PAH degradation pathway |
| *Mycobacterium smegmatis* str. MC2 155 | Non-pathogenic species |
| *Mycobacterium ulcerans* Agy99 | Causes Buruli ulcer in humans |
| *Mycobacterium vanbaalenii* PYR-1 | Non-pathogenic strain, useful for bioremediation |
| *Mycobacterium yongonense* 05-1390 | Pulmonary disease |
| *Mycobacterium arupense* | Tenosynovitis and osteoarticular infections |
| *Mycobacterium thermoresistibile* ATCC 19527 | Human pathogen |
| *Mycobacterium aromaticivorans* | Powerful degrading capacity to polycyclic aromatic compounds (PAHs) |
| *Mycobacterium asiaticum* | *pneumonia*, human infection |
| *Mycobacterium bohemicum* | Human, Veterinary  Down's syndrome and tuberculosis |
| *Mycobacterium caprae* | Tuberculosis among animals and to a limited extent in humans |
| *Mycobacterium colombiense* | Respiratory disease and disseminated infection in immunocompromised HIV patients as well as lymphadenopathy in immunocompetent children |
| *Mycobacterium conceptionense* | Opportunistic pathogen. Infections include skin and soft tissue infection characterized by slowly progressive granulomatous inflammation, lymphadenitis, skeletal and pulmonary infections, and catheter-related, disseminated infection in immunocompromised patients |
| *Mycobacterium cosmeticum* | Cosmetic infection and from a nail salon |
| *Mycobacterium europaeum* | Higher risk of lung infection in immunocompromised hosts/ patients |
| *Mycobacterium fortuitum* | Occasionally cause miscellaneous human infections, including skin and soft tissue infections, post-surgical wound infections, lymphadenitis, and catheter-related infections, but it can also cause lung disease. |
| *Mycobacterium genavense* | Disseminated infection, opportunistic [pathogen](https://en.wikipedia.org/wiki/Pathogen), gastro-intestinal disorders |
| *Mycobacterium hassiacum* | Non-pathogenic |
| *Mycobacterium heraklionense* | A chronic tenosynovitis associated with trauma and foreign body introduction in an otherwise healthy individual |
| *Mycobacterium immunogenum* | Implicated in cutaneous infection in both healthy and immunosuppressed patients |
| *Mycobacterium iranicum* | Infrequent human pathogen |
| *Mycobacterium kyorinense* | Pathogenic for humans and have substantial clinical effects |
| *Mycobacterium lentiflavum* | Infections involve in skin or lymph node. Also, it has been isolated from pleural effusions, ascites, and from lung tissues, chronic pulmonary infection in immunodominant patients |
| *Mycobacterium lepromatosis* | Lepromatous leprosy (LL) and diffuse lepromatous leprosy (DLL) |
| *Mycobacterium llatzerense* | Abdominal abscess |
| *Mycobacterium mageritense* | Pneumonia in an immunocompromised patient. |
| *Mycobacterium kansasii* ATCC 12478 | Pulmonary disease in immuno-  compromised individual, found in aquatic environment |
| *Mycobacterium nebraskense* | Nodular pulmonary disease |
| *Mycobacterium neoaurum* | Bloodstream infection is rare and occurs most often in immunocompromised hosts who present with undifferentiated fever and have an indwelling venous catheter |
| *Mycobacterium obuense* | Capable of forming a black product from p-aminosalicylate and salicylate.  Heat-killed *Mycobacterium obuense* is immunomodulatory and has been used to direct the immune response in the treatment of cancers - notably pancreatic cancer and malignant melanoma |
| *Mycobacterium orygis* | Causative agent of tuberculosis in animals and humans |
| *Mycobacterium parascrofulaceum* | Opportunistic pathogen, pulmonary infection |
| *Mycobacterium rhodesiae* | Pulmonary disease |
| *Mycobacterium senegalense* | Cause disease among cattle in east Africa |
| *Mycobacterium setense* | Traumatic chronic skin abscess associated with osteitis |
| *Mycobacterium septicum* | In the immunosuppressed patient, these organisms cause serious infections such as catheter-related bacteraemia or disseminated disease |
| *Mycobacterium simiae* | lymphadenitis in an immunocompetent children, pediatric case, mostly in  pulmonary and reticuloendothelial system |
| *Mycobacterium arupense* | Pulmonary infection and tenosynovitis |
| *Mycobacterium triplex* | Causes episodic in immunocompetent host |
| *Mycobacterium tusciae* | Cervical lymphadenitis in immunocomponent host |
| *Mycobacterium vaccae* | Low pathogenicity for humans, heat-killed M. vaccae immunotherapeutic agent |
| *Mycobacterium vulneris* | Non-tuberculosis opportunistic pathogen |
| *Mycobacterium xenopi* | Pulmonary infection |
| *Mycobacterium gastri 'Wayne'* | Reports of pediatric infection,  casual resident of human stomachs |
